# Supplementary material for: Enriched Alternative Splicing in Islets of Diabetes-Susceptible Mice
Source: Int J Mol Sci. 2021 Aug 10;22(16):8597. doi: 10.3390/ijms22168597 (PMC8395343; doi:10.3390/ijms22168597)
Supplement: Supplementary file 1 [file ijms-22-08597-s001.zip › supplementary figures.pdf]

*Supplementary Materials*

# Enriched alternative splicing in islets of diabetes-susceptible mice

**Ilka Wilhelmi [1,2], Alexander Neumann [3], Markus Jähnert [1,2], Meriem Ouni [1,2,†] and Annette Schürmann [1,2,4,5,*,†]**

[1] Department of Experimental Diabetology, German Institute of Human Nutrition (DIfE), Potsdam, Germany; Ilka.wilhelmi@dife.de (I.W.); Markus.jaehnert@dife.de (M.J.); Meriem.ouni@dife.de (M.O.)
[2] German Center for Diabetes Research (DZD), Munich, Germany
[3] Omiqa Bioinformatics, Berlin, Germany; Alexander.neumann@omiqa.bio
[4] University of Potsdam, Institute of Nutritional Sciences, Nuthetal, Germany
[5] Faculty of Health Sciences, joint Faculty of the Brandenburg University of Technology Cottbus—Senftenberg, the Brandenburg Medical School Theodor Fontane and the University of Potsdam, Germany
† These authors contributed equally to this work.
* Correspondence: schuermann@dife.de

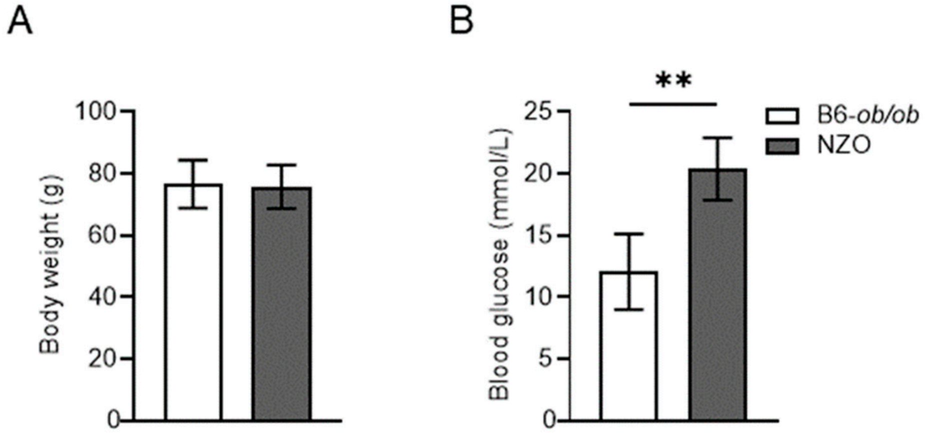


**Supplemental Figure S1. A** Body weight of B6-*ob/ob* and NZO mice at the day of sacrifice (age of 18 weeks). **B** Blood glucose levels of B6-*ob/ob* and NZO mice at the day of sacrifice. Data is shown as mean ± SD from 6 mice per genotype. * $p \leq 0.05$, student's t-test with Welch's correction.

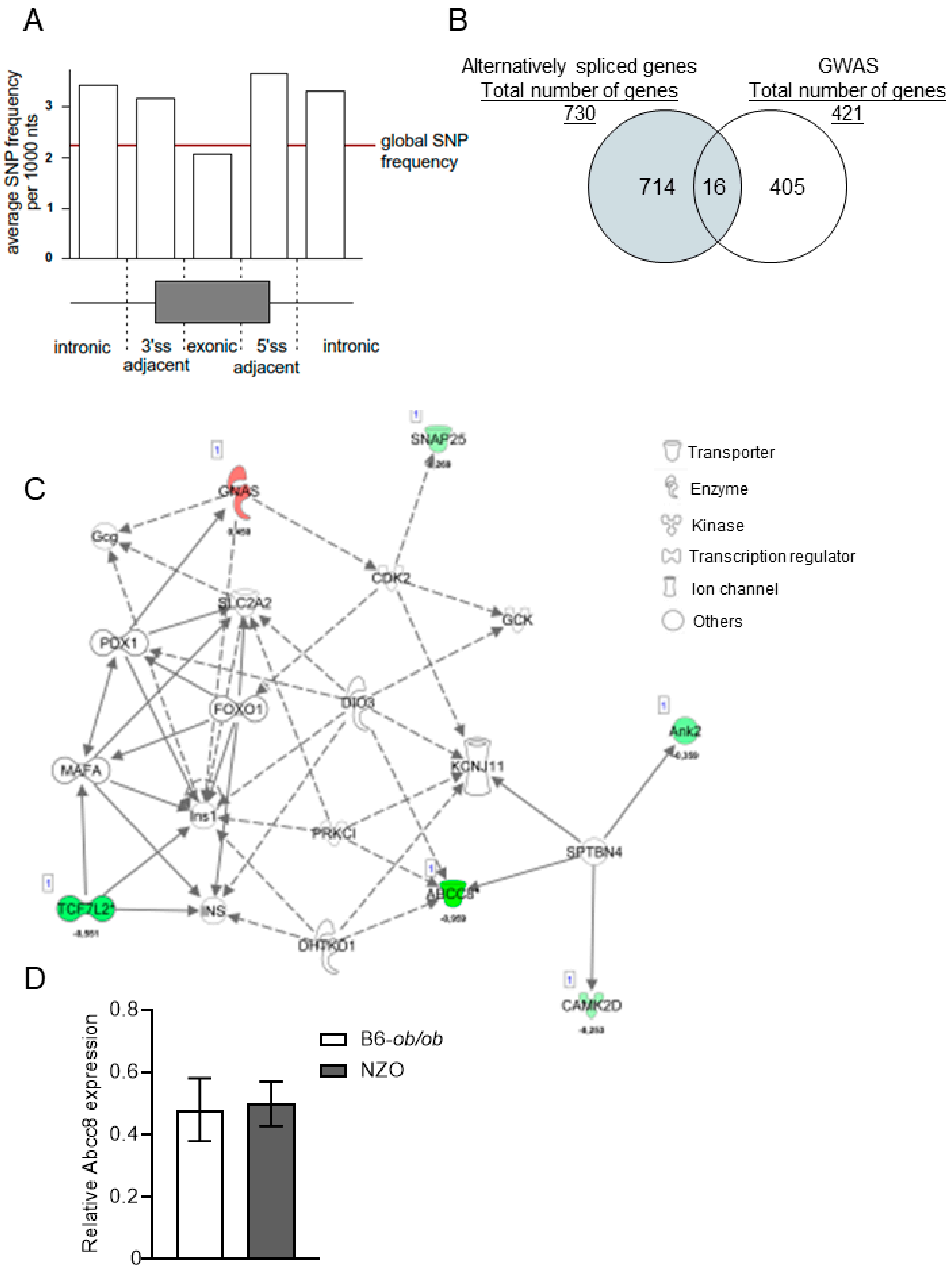


**Supplemental Figure S2. A** SNP frequency at indicated positions adjacent to alternative exons. Red line represents global SNP frequency. **B** Venn diagram showing the overlay of differentially spliced genes in islets of diabetes susceptible mice and human T2D genes identified by GWAS. **C** Network of genes with alternatively spliced cassette exons generated by Ingenuity Pathway Analysis (IPA). Green = negative ΔPSI meaning more exon skipping in islets from NZO mice, red = positive ΔPSI. **D** Relative expression of *Abcc8* in islets from male B6-*ob/ob* and NZO mice treated as described in Figure 1A (n = 4). Data is shown as mean ± SD, * $p \leq 0.05$, student's t-test with Welch's correction.
